# IL-1β-induced activation of p38 promotes metastasis in gastric adenocarcinoma via upregulation of AP-1/c-fos, MMP2 and MMP9

**DOI:** 10.1186/1476-4598-13-18

**Published:** 2014-01-31

**Authors:** Qiaojia Huang, Fenghua Lan, Xiaoting Wang, Yinghao Yu, Xuenong Ouyang, Feng Zheng, Junyong Han, Youdong Lin, Yanchuan Xie, Feilai Xie, Wei Liu, Xiaoli Yang, Han Wang, Lihong Dong, Lie Wang, Jianming Tan

**Affiliations:** 1 Department of Experimental Medicine, Fuzhou General Hospital (Dongfang Hospital), 156 North Xi-er Huan Road, Fuzhou City, Fujian Province 350025, China; 2 Department of Pathology, Fuzhou General Hospital (Dongfang Hospital), 156 North Xi-er Huan Road, Fuzhou City, Fujian Province 350025, China; 3 Department of General Surgery, Fuzhou General Hospital (Dongfang Hospital), 156 North Xi-er Huan Road, Fuzhou City, Fujian Province 350025, China; 4 Department of Oncology, Fuzhou General Hospital (Dongfang Hospital), 156 North Xi-er Huan Road, Fuzhou City, Fujian Province 350025, China; 5 Department of Nephrology, Fuzhou General Hospital (Dongfang Hospital), 156 North Xi-er Huan Road, Fuzhou City, Fujian Province 350025, China; 6 Organ Transplant Institute, Fuzhou General Hospital (Dongfang Hospital), 156 North Xi-er Huan Road, Fuzhou City, Fujian Province 350025, China

**Keywords:** IL-1β, p38, Gastric adenocarcinoma, MMP2 and MMP9, AP-1, Metastasis

## Abstract

**Background:**

Interleukin-1β (IL-1β) has been implicated in the progression of gastric adenocarcinoma (GA); however, the molecular mechanisms of action of IL-1β in GA are poorly characterized. P38 and JNK are the major MAPK family members that regulate IL-1β signaling pathways. Here, we investigated the role of both p38 and JNK in IL-1β-induced GA cell migration, invasion and metastatic potential.

**Methods:**

The effects of IL-1β-induced p38 and JNK activation in GA cells were determined using in vitro Transwell migration and invasion assays of MKN-45 and AGS cells, or an in vivo metastasis assay in nude mice. The IL-1β-induced p38 signaling pathway was further characterized in GA cells. Activation of the IL-1β/p38 signaling pathway was also assessed in human primary GA tissues by immunohistochemistry.

**Results:**

IL-1β-induced activation of p38 increased GA cell migration and invasion in vitro and promoted the metastatic potential of GA cells in vivo; these effects were attenuated by p38 siRNA or the p38 inhibitor SB202190. MMP2 or MMP9 siRNAs and the MMP2/9 inhibitor BiPS also inhibited IL-1β-induced GA cell migration and invasion in vitro. IL-1β-induced p38 activation significantly increased MMP2 and MMP9 mRNA and protein expression and activity. Luciferase reporter assays demonstrated that the activator protein-1 (AP-1) and the AP-1 binding sites of the *MMP9* promoter (−670/MMP9) were activated by IL-1β-induced p38 activation. Phospho-p38 was significantly upregulated in human GA tissues (compared to matched non-neoplastic tissues), and significantly associated with lymph node metastasis, and invasion beyond the serosa. Expression of phospho-p38 significantly correlated with IL-1β, MMP2, MMP9, and c-fos expression in both human GA tissues and GA cell metastases in the lungs of nude mice. IL-1β was also capable of activating JNK in GA cells, but activation of JNK was not associated with GA cell migration and invasion. Therefore, IL-1β-induced the migration and invasion in GA cells were regulated by p38, but not by JNK.

**Conclusions:**

IL-1β-induced p38 activation and the IL-1β/p38/AP-1(c-fos)/MMP2 & MMP9 pathway play an important role in metastasis in GA; this pathway may provide a novel therapeutic target for GA.

## Background

Increasing evidence indicates that tumors are promoted and sustained by inflammatory signals from the tumor microenvironment, and the tumor microenvironment plays important roles in the promotion of cancer [1,2]. Cytokines, especially the cytokines secreted by tumor cells, are essential components of the tumor microenvironment. Tumor necrosis factor alpha (TNF-α), interleukin-1β (IL-β) and IL-6 are the most well-characterized cytokines which have been demonstrated to be closely related to cancer progression. A lot of studies have shown that inflammation induced by cytokines plays an important role in the development of gastric cancer [3]. It is well established that infections, especially those induced by *Helicobacter pylori,* are capable of inducing gastric mucosal inflammatory responses, resulting in upregulation of IL-1β, which in turn may promote inflammation-associated carcinogenesis [4]. However, the underlying molecular mechanisms for the role of IL-1β signaling in gastric carcinogenesis remain largely unknown, and are currently of interest.

P38 is a member of the mitogen-activated protein kinase (MAPK) superfamily. The MAPK signaling pathways have been well investigated, and are comprised of at least three superfamilies of MAPKs which regulate diverse cellular activities [5]. It is well known that p38 MAPK is capable of regulating a lot of cellular responses to cytokines and stress, including IL-1β [6]; however, recent data demonstrated that p38 is also closely related to the development of different types of human cancer via its ability to elevate cancer cell migration and invasion in response to various stimuli, including inflammatory factors [6]. Additionally, p38 is also involved in the regulation of cell differentiation and apoptosis. Four isoforms of p38 have been identified so far: p38-α, p38-β, p38-γ, and p38-δ [7]. Though the amino acid sequences of these p38 MAPKs are mostly identical, the expression pattern of each isoform varies [8]. P38-α is the major p38 MAPK and is expressed ubiquitously, p38-β is mainly expressed in the brain, whereas p38γ is abundantly expressed in skeletal muscle [9] and p38-δ is mainly expressed in endocrine glands [10]. Many studies have also demonstrated that p38 participates in IL-1β signaling cascades in a set of cell types, especially in mouse embryonic fibroblast (MEF) cells and macrophages cells [11,12]; however, very little is known about the function of IL-1β-activated p38 in gastric cancer.

c-Jun N-terminal kinase (JNK) is another MAPK family member which is also well known to play an important role in regulation IL-1β signaling pathway [13]. In addition to participation in regulation inflammatory signal pathway, JNK performs several other important cellular functions including regulation of cell growth, differentiation, survival and apoptosis. Furthermore, recent studies demonstrate that JNK is frequently over-expressed in different cancer tissues, and up-regulation of JNK may be closely associated with cancer invasion [14]; however, whether JNK participates in regulation of IL-1β-induced gastric cancer cell migration and invasion remains largely unknown.

Gastric adenocarcinoma (GA) is the most common neoplastic tumor of the stomach; therefore, we focused on GA in this study. Here, we investigated the activation of p38 and JNK in response to IL-1β, and their effect on IL-1β-induced metastatic potential of GA cells in vitro or vivo*.* Additionally, the expression of phospho-p38 (p-p38) in GA, its relationship to the clinicopathologic features of GA, and the correlation between the expression of IL-1β and p-p38 were investigated in human paraffin-embedded GA tissues using immunohistochemistry. Finally, we also characterized the molecular mechanisms which regulate the IL-1β-induced p38-mediated metastatic potential of GA cells.

## Results

### IL-1β-induced activation of p38 promotes GA cell migration and invasion in vitro

**Figure 1:**
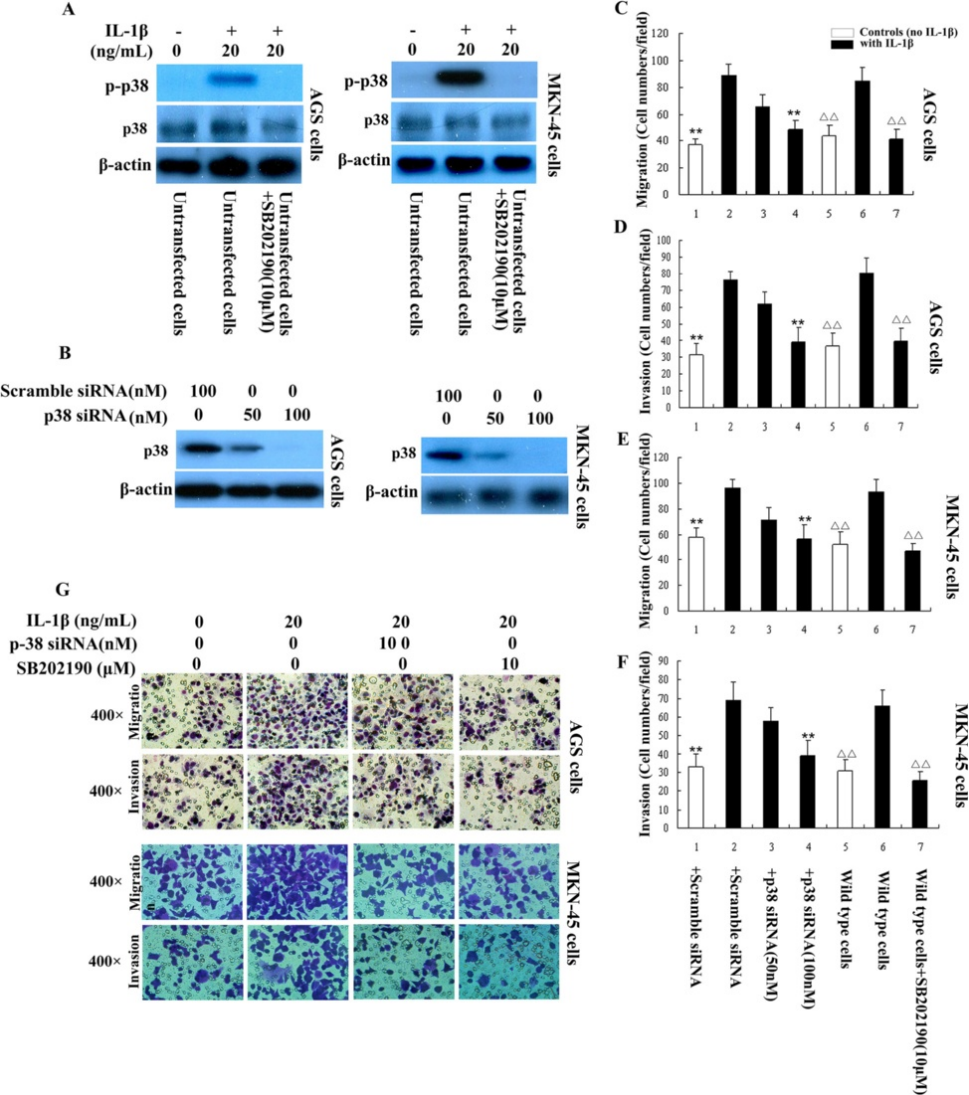
**IL-1β promotes GA cell migration and invasion by activating p38. A**: Western blots confirmed that p-p38 could be induced by 30 min stimulation with IL-1β in AGS and MKN-45 cell lines; the activation of p38 by IL-1β was inhibited by p38 inhibitor SB202190. **B**: Transfection of p38 siRNA knocked down p38 expression in both the two GA cell lines. **C-F**: Treatment of GA cells with IL-1β increased cell migration and invasion in vitro; these effects were inhibited by p38 siRNA or the p38 pathway inhibitor SB202190. ^**^P < 0.05 vs. scramble siRNA (Control siRNA)-transfected cells stimulated with IL-1β; ^**△△**^*P* < 0.05 vs. untransfected cells (wild type cells) stimulated with IL-1β. Bars indicate the mean ± SD number of cells per field of view (× 400 magnification) in the migration and invasion assays. **G**: Representative light microscope images of AGS and MKN-45 cell migration and invasion in the Transwell assays.

First, we investigated whether IL-1β was able to activate p38 signaling in GA cells. As shown in Figure 1, activation of p38 (p-p38) was detected in both GA cell lines (AGS and MKN-45 cells) after treatment with IL-1β for 30 min; IL-1β-induced activation of p38 was inhibited by the p38 inhibitor SB202190 (Figure 1A).

P38 can promote the migration and invasion of different cancer cells [15]. To investigate whether IL-1β can promote the migration and invasion of GA cells via activating p38 signaling, GA cells transfected with a scrambled siRNA (control siRNA) or p38 siRNA, or GA cells pre-treated with or without the p38 pathway inhibitor SB202190 were stimulated with IL-1β. Transwell migration and invasion assays demonstrated that IL-1β stimulation increased the migration and invasion of both AGS and MKN-45 cells; however, IL-1β-induced GA cell migration and invasion were significantly attenuated by knockdown of p38 using siRNA (Figure 1B to G) or pretreatment with SB202190 (Figure 1C to G). Taken together, these data strongly suggest that IL-1β promoted GA cell migration and invasion are mediated by p38.

### IL-1β-induced activation of p38 upregulates MMP2 and MMP9 expression and activity in GA cells

**Figure 2:**
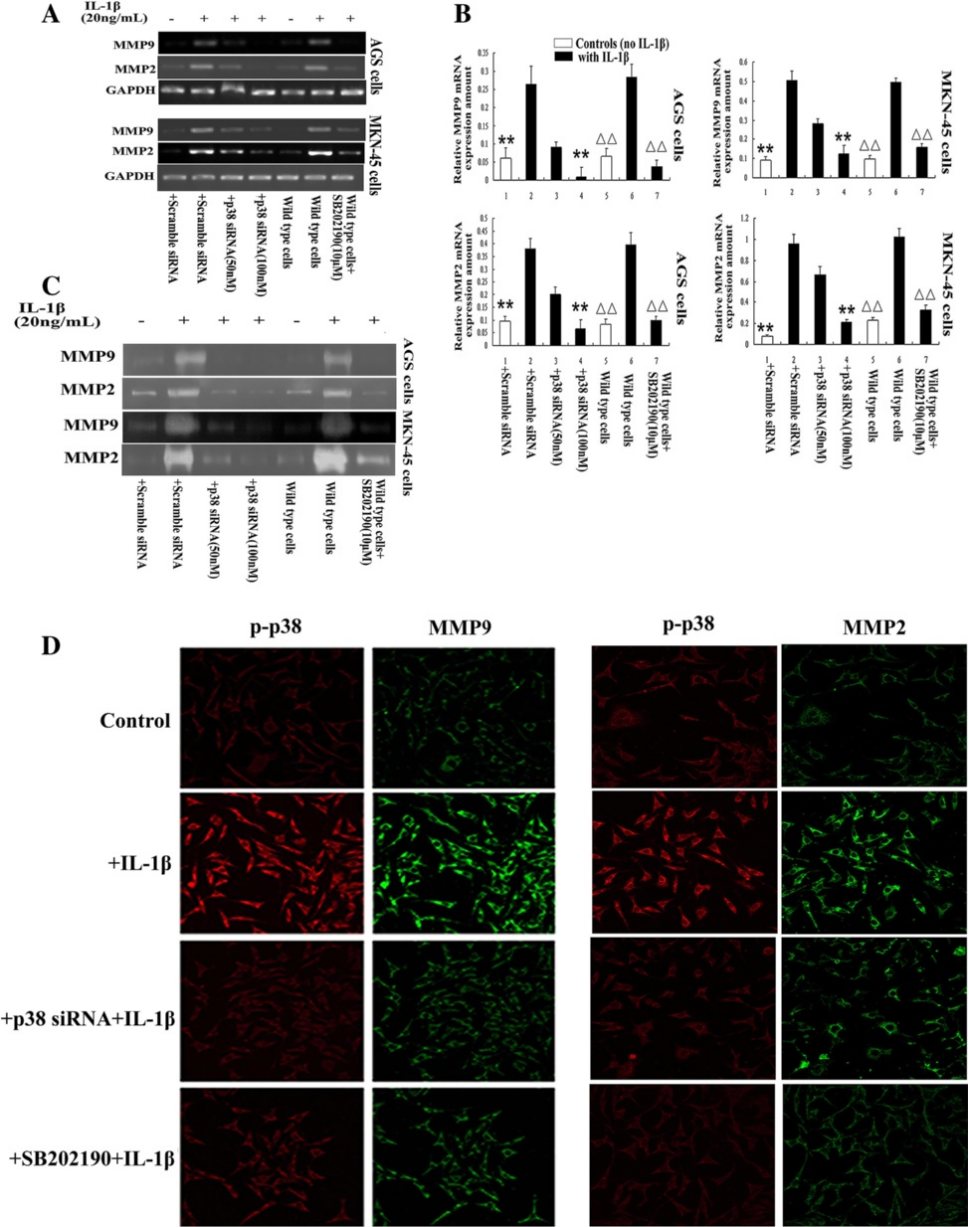
**IL-1β upregulates MMP2 and MMP9 expression and activity by activating p38. A**: RT-PCR analysis showed that *MMP2* and *MMP9* mRNA were upregulated in both AGS and MKN-45 cells in response to treatment with IL-1β; this effect was blocked by p38 siRNA or the p38 inhibitor SB202190. **B**: Quantification of the expression of *MMP2* and *MMP9* mRNA normalized to *GAPDH* mRNA. ^**^P < 0.05 vs. scramble siRNA-transfected cells stimulated with IL-1β. ^**△△**^*P* < 0.05 vs. untransfected cells stimulated with IL-1β. **C:** Gel zymography showed that MMP2 and MMP9 activity were upregulated in both AGS and MKN-45 cells in response to treatment with IL-1β; this effect was blocked by p38 siRNA or the p38 inhibitor SB202190. **D:** Immunocytochemical staining and confocal microscopy confirmed that IL-1β increased activation of p38 (red), and MMP2 and MMP9 (green) expression in MKN-45 GA cells; these effects were blocked by p38 siRNA or the p38 inhibitor SB202190.

MMP2 and MMP9 have been shown to play important roles in cancer cell invasion and metastasis [16]. To explore whether MMP2 and MMP9 also participate in the IL-1β-induced p38-regulated metastatic potential of GA cells, RT-PCR, immunocytochemistry and confocal microscopy, and the zymography assay were carried out to determine MMP2 and MMP9 expression and activity in GA cell lines transfected with control siRNA or p38 siRNA, or pretreated with or without the p38 inhibitor SB202190, and then treated with or without IL-1β. As expected, MMP2 and MMP9 expression and activity were elevated in response to IL-1β treatment (Figure 2A to C). Knockdown of p38 using siRNA, or pretreatment with the p38 inhibitor SB202190 significantly decreased Il-1β-induced MMP2 and MMP9 mRNA expression and activity in both GA cell lines (Figure 2A to C).

Immunocytochemistry and confocal microscopy demonstrated that p-p38 was weakly expressed in untreated MKN-45 cells, which also expressed very low levels of MMP2 and MMP9. After stimulation with IL-1β, significantly increased levels of p-p38, MMP2 and MMP9 were detected in the MKN-45 cells; these IL-1β-induced effects were inhibited by p38 siRNA and SB202190 (Figure 2D). Taken together, these results strongly suggest that the IL-1β through p38-induced invasion and migration of GA cells is mediated via the ability of p-p38 to upregulate MMP2 and MMP9 expression and activity.

### IL-1β-induced activation of p38 upregulates MMP2 and MMP9 by activating AP-1-dependent transcription in GA cells

**Figure 3:**
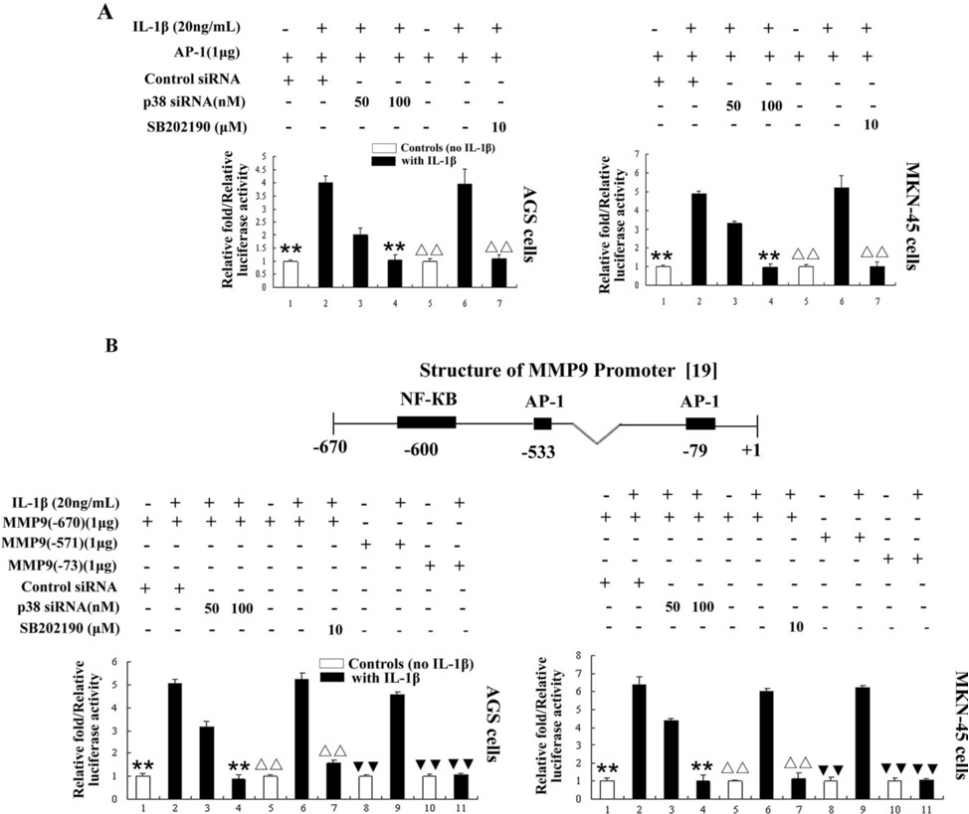
**IL-1β activates AP-1 activity through p38 pathway. A**: Both GA cells were transfected with AP-1 reporter plasmid or AP-1 reporter plasmid together with scramble siRNA or p38 siRNA. AP-1 luciferase reporter gene activity was significantly increased by IL-1β stimulation in both AGS and MKN-45 cells; these effects were significantly inhibited by p38 siRNA in a dose-dependent manner and also inhibited by the p38 inhibitor SB202190. ** *P* < 0.05 vs. control siRNA (scramble siRNA) + AP-1 luc-transfected cells stimulated with IL-1β; ^ΔΔ^*P* < 0.05 vs. AP-1 luc-transfected cells stimulated with IL-1β; relative luciferase activity was normalized to B-gal. **B**: IL-1β-induced p38-mediated activation of the *MMP9* promoters is dependent on the AP-1-binding sites. ** *P* < 0.05 vs. transfected −670/MMP9 + control siRNA cells stimulated with IL-1β; ^ΔΔ^*P* < 0.05 vs. transfected −670/MMP9 cells stimulated with IL-1β; ^▼▼^*P* < 0.05 vs. transfected −570/MMP9 cells stimulated with IL-1β; relative luciferase activity was normalized to B-gal.

It is well documented that the transcription factor activator protein-1 (AP-1) can regulate the expression of MMP2 and MMP9 [17], and activation of p38 is able to regulate AP-1 activation [18]. In order to examine whether IL-1β-induced p38-mediated elevated MMP2 and MMP9 expression and activity are dependent on AP-1, the activation of AP-1-dependent transcription was investigated in GA cells treated with or without IL-1β, in the presence or absence of p38 inhibition, using an AP-1 luciferase reporter assay. IL-1β increased the activity of the AP-1 in both GA cell lines (Figure 3A); however, inhibition of p38 using p38 siRNA or pretreated cells with p38 inhibitor SB202129 reduced IL-1β-induced AP-1 activity in both GA cell lines (Figure 3A). These results indicate that IL-1β induced, p38 mediated expression of MMP2 and MMP9 are dependent on AP-1.

In order to further confirm the role of AP-1 in IL-1β-induced p38 pathway, luciferase reporter gene vectors containing the AP-1 sites of the *MMP9* promoter regions were transfected into the GA cells. In accordance with the AP-1 reporter gene assays, the luciferase activities of the −670/*MMP9* promoter region (containing two AP-1 binding sites [−533 and −79] and one NF-КB binding site [−600] [19-21]) significantly increased in IL-1β-stimulated cells (Figure 3B). Transfection of the cells with p38 siRNA or pretreated cells with p38 inhibitor SB202129 reduced the IL-1β-induced luciferase activity of the −670/*MMP9* promoter reporter gene (Figure 3B). The luciferase activity of the *MMP9* promoter (-571/*MMP9*) was not altered by deletion of the NFκB-binding site (−600). Furthermore, when the AP-1 sites (−79 and −533) of the *MMP9* promoter were deleted (−73/*MMP9* mutants), the luciferase activity of the reporter gene significantly decreased, compared to the respective wild-type control reporter genes (Figure 3B). Collectively, these data strongly indicate that IL-1β induces activation of the p38 signaling pathway, which promotes the invasion and migration of GA cells via AP-1-dependent upregulation of MMP2 and MMP9 expression and activity.

### Knockdown of MMP2 or MMP9 decreases IL-1β-induced migration and invasion in GA cells

**Figure 4:**
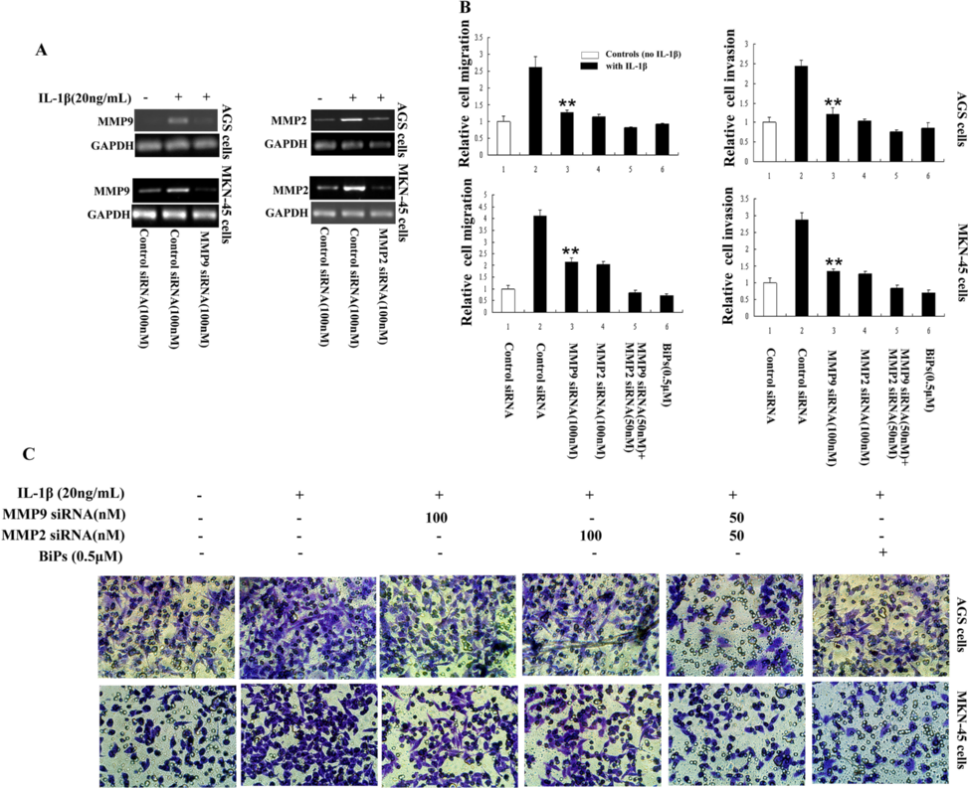
**Knockdown of *MMP2 *or *MMP9 *or pretreatment with the MMP2/9 inhibitor BiPs inhibits IL-1β-induced GA cell migration and invasion in vitro. A**: RT-PCR confirmed the efficiency of the MMP2 or MMP9 siRNAs, which significantly reduced *MMP2* or *MMP9* expression, respectively, by up to more than 75%. **B**: IL-1β increased GA cell migration and invasion in vitro; these effects were inhibited by MMP2 or MMP9 siRNA or the MMP2/9 inhibitor BiPs. ^**^P < 0.05 vs. scramble siRNA (control siRNA)-transfected cells stimulated with IL-1β. Bars are the mean ± SD fold changes normalized to control group in the migration and invasion assays. **C**: Representative light microscopy images of AGS and MKN-45 cell migration and invasion in the Transwell migration and invasion assays.

To further confirm that IL-1β-induced GA cell migration and invasion are associated with upregulation of MMP2 and MMP9, AGS and MKN-45 cells were transfected with siRNAs against *MMP2* or *MMP9*, or pretreated with or without the MMP2/MMP9 inhibitor BiPS, and then stimulated with IL-1β. The Transwell migration and invasion assays demonstrated that the IL-1β-induced migration and invasion of GA cells were significantly attenuated by knockdown of *MMP2* or *MMP9,* or pre-treatment with BiPS, compared to control cells (Figure 4A to C). Therefore, upregulation of MMP2 and MMP9 are crucial for IL-1β-induced GA cell migration and invasion.

### IL-1β-induced activation of JNK doesn’t participate in regulation of GA cell migration and invasion

**Figure 5:**
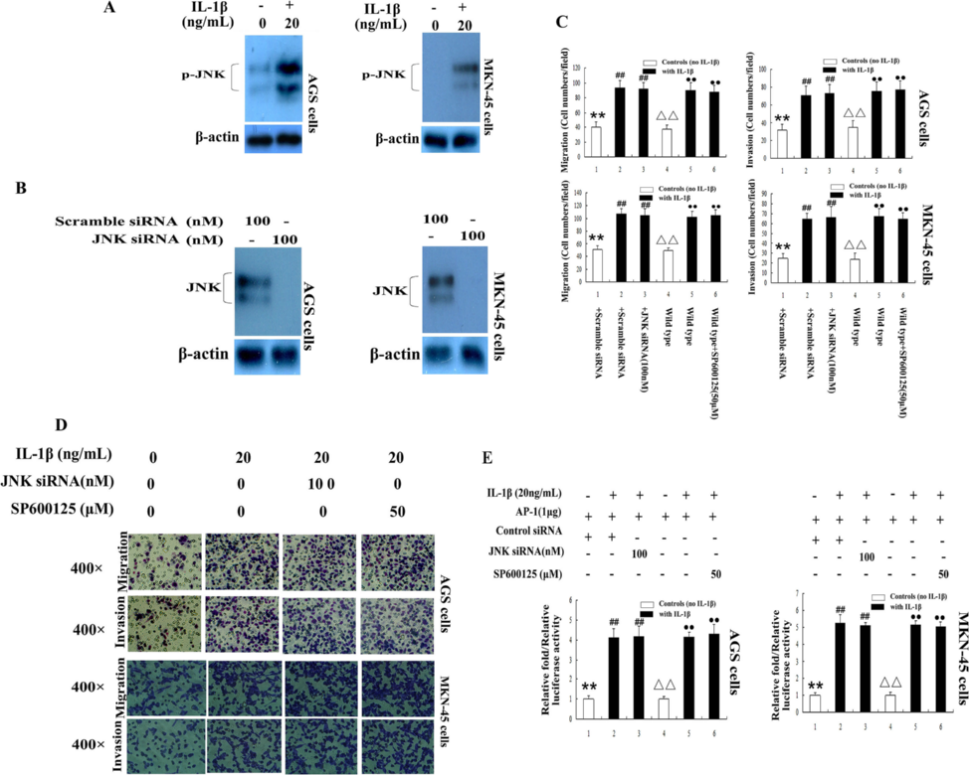
**The activation of JNK is not associated with IL-1β-induced GA cell migration and invasion. A**: p-JNK was detected by 30 min stimulation with IL-1β in AGS and MKN-45 cell lines. **B**: Transfection of JNK siRNA knocked down JNK expression in both the two GA cell lines. **C-D**: Treatment of GA cells with IL-1β increased cell migration and invasion in vitro; these effects were not inhibited by JNK siRNA nor the JNK pathway inhibitor SP600125. ^**^P < 0.05 vs. scramble siRNA-transfected cells stimulated with IL-1β; ^**△△**^*P* < 0.05 vs. untransfected cells (wild type cells) stimulated with IL-1β; ^##^ or ^●●^ no significant different between these two groups was detected (*P*>0.05). Bars indicated the mean ± SD number of cells per field of view in the migration and invasion assays. **D:** Representative light microscope images of AGS and MKN-45 cell migration and invasion in the Transwell assays. **E:** Both GA cells were transfected with AP-1 reporter plasmid or AP-1 reporter plasmid together with scramble siRNA or JNK siRNA. AP-1 luciferase reporter gene activity was significantly increased by IL-1β stimulation in both AGS and MKN-45 cells; these effects were not inhibited by JNK siRNA nor inhibited by the JNK inhibitor SP600125 neither. ** *P* < 0.05 vs. control siRNA (scramble siRNA) + AP-1 luc-transfected cells stimulated with IL-1β; ^ΔΔ^*P* < 0.05 vs. AP-1 luc-transfected cells stimulated with IL-1β; ^##^ or ^●●^ no significant different between these two groups was detected (*P*>0.05). Relative luciferase activity was normalized to B-gal.

It is well known that members of MAPK play important roles in regulation of cellular responses to cytokines and stress, and P38 and JNK are the major MAPK family members that regulate IL-1β signaling pathways. To understand whether JNK is also associated with IL-1β-induced GA cell migration and invasion, Western blot analysis was performed to detect the activation of JNK in response to IL-1β. As exhibited in Figure 5A, p-JNK was detected in both AGS and MNK-45 cell lines after stimulation with IL-1β for 30 min. However, the results of both Transwell migration and invasion assays showed that the increased migration and invasion of both AGS and MKN-45 cells induced by IL-1β stimulation were not attenuated by knockdown JNK with siRNA nor attenuated by inhibition JNK pathway with JNK inhibitor SP600125 neither (Figure 5B to D); The number of migrated and invasive cells almost did not showed change before or after transfection with siRNA against JNK (P>0.05) or with or without pre-treated with JNK inhibitor SP600125 (P>0.05). JNK was not associated with IL-1β-promoted the GA cell migration and invasion having been further verified by AP-1 luciferase reporter assay. As the upstream kinase of c-jun (another important component of AP-1), JNK is able to activate AP-1, and the activation of AP-1 by JNK is closely related with JNK’s function on regulation of various cellular reaction including cancer cell migration and invasion [22]; however, IL-1β-induced AP-1 activation in both AGS and MKN-45 cells was not inhibited by JNK siRNA nor JNK inhibitor SP600125 neither (Figure 5E). All together, these data strongly indicate that the increased GA migration and invasion promoted by IL-1β are not regulated by JNK.

### Phospho-p38 is upregulated and correlates with the expression of IL-1β, MMP2, MMP9 and c-fos in human GA tissues

**Figure 6:**
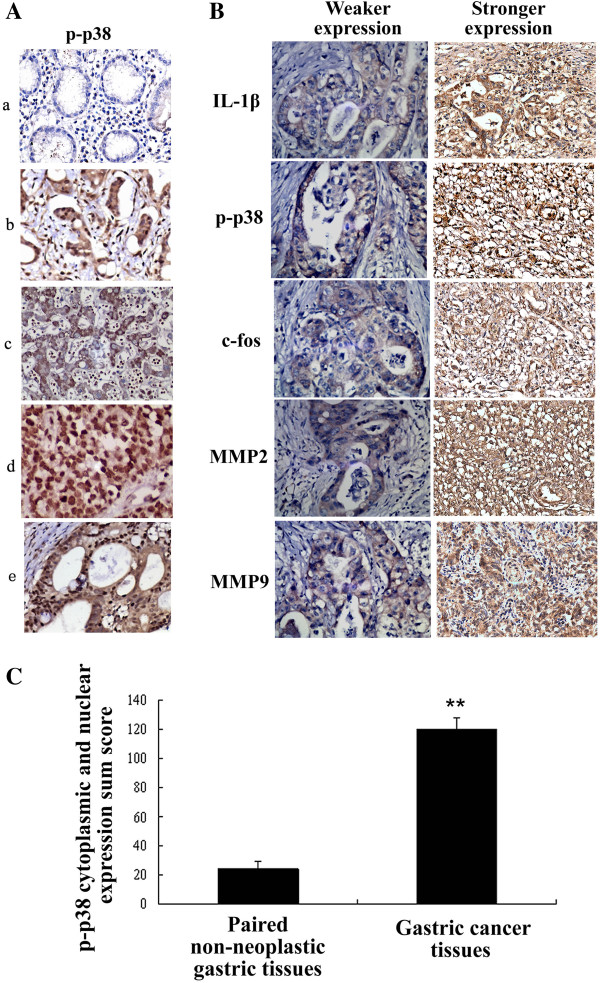
**Phosphorylated p38 is overexpressed in human GA, and the expression of p-p38 positively correlates with IL-1β, MMP2, MMP9 and c-fos expression in GA tissues. A**: Overexpression of p-p38 was frequently observed in GA tissues, compared to the paired non-neoplastic gastric tissues. **a**, P-p38 was not detected or only weakly expressed in non-neoplastic gastric tissues. **b** to **e**: different intensities of positive p-p38 expression in GA tissues. **B**: Expression of p-p38 positively correlated with IL-1β, MMP2, MMP9, and c-fos expression in human GA tissues. GA tissue sample exhibiting stronger IL-1β expression and stronger p-p38, MMP2, MMP9 and c-fos expression; and weaker IL-1β expression and weak p-p38, MMP2, MMP9 and c-fos expression. **C**: The sum scores of positive staining intensity of IHC for p-p38. ** *P* < 0.05 vs. paired non- neoplastic gastric tissues.

The expression of p-p38 in a series of 105 GA tissues and the paired non-neoplastic gastric tissues was examined by immunohistochemistry (IHC). Of the 105 cancer samples, 53 cases of GA tissues (50.48%) exhibited over-expression of p-p38 compared to the paired non-neoplastic gastric tissues (*P* < 0.05; Figure 6A). Positive p-p38 expression was frequently observed in both the GA cell cytoplasm and nucleus.

No significant associations were observed between overexpression of p-p38 in the patients’ age (< 50 or ≥ 50 years), gender, tumor size (< 3 cm or ≥ 3 cm), histological type, or grade of differentiation. However, overexpression of p-p38 displayed significantly related with lymph node metastasis (*P* < 0.05), and invasion beyond the serosa (*P* < 0.05). These data suggest that overexpression of p-p38 is associated with metastasis in human GA.

As exhibited in Figure 6B, the expression of p-p38 showed good correlativity with the levels of IL-1β, MMP2, MMP9 and AP-1(c-fos) in GA tissue, and a significant correlation between the elevated p-p38 expression and upregulation of IL-1β, MMP2, MMP9 and c-fos in GA tissue (r = 0.72, p<0.01; r = 0.63, p<0.01; r = 0.58, p<0.05 and r = 0.69, p<0.01) was detected when analyzed by Spearman method.

The sum scores of positive staining intensity of IHC for p-p38 in both 105 cases of GA tissues and paired non- neoplastic gastric tissues were exhibited in Figure 6C.

### Invasion assay in nude mice

MKN-45 cells transfected with a scrambled siRNA or p38 siRNA were injected into the tail vein of BALB/c nu/nu mice; IL-1β or PBS were also intraperitoneally injected from the day of the cells were injected for 14 days. Group 1 were injected with PBS and scrambled siRNA-transfected MKN-45 cells; group 2 were injected with IL-1β and scrambled siRNA-transfected MKN-45 cells; and group 3 were injected with p38 siRNA-transfected MKN-45 cells and IL-1β.

**Figure 7:**
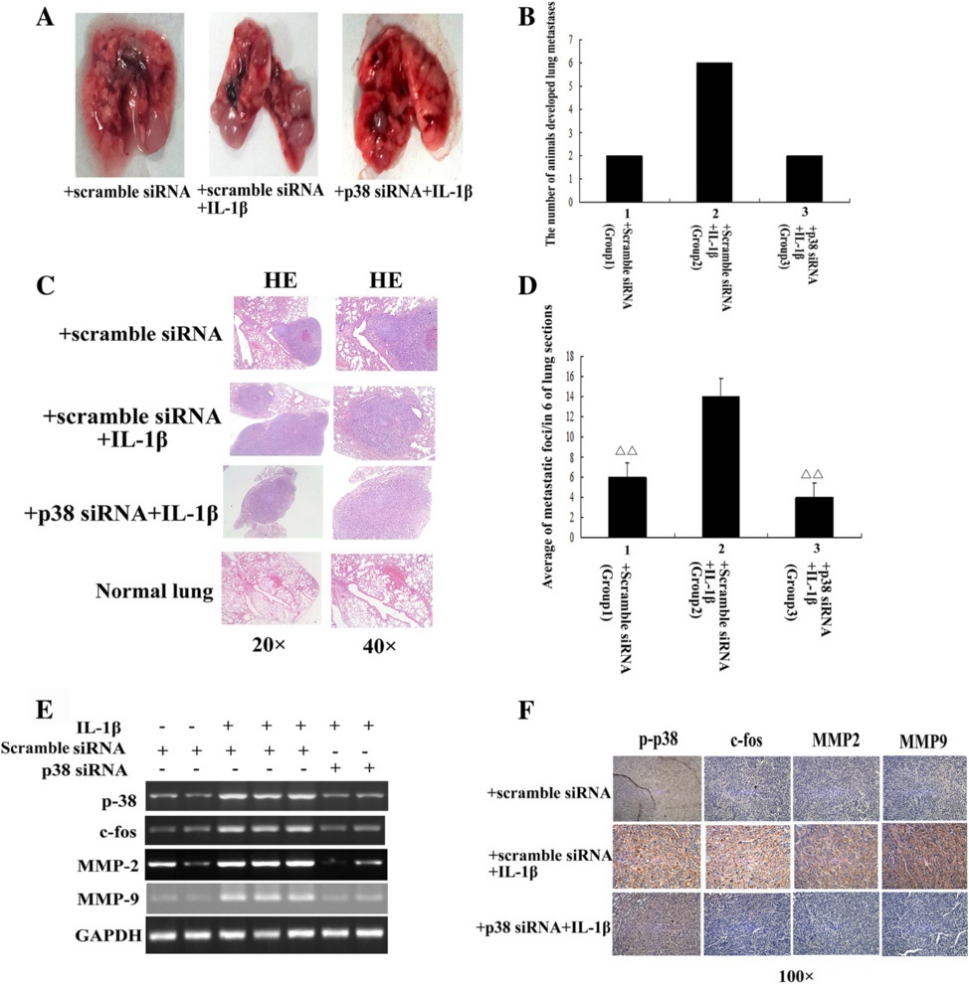
**IL-1β stimulation increases the metastatic potential of GA cells in vivo via a p38–dependent mechanism. A**: Representative lung metastases formed in different group mouse. **B**: Bars indicated the number of animals developed lung metastases. **C**: Representative results of HE staining of lung metastases. **D**: Bars indicated the mean ± SD number of metastatic foci in 6 of lung sections/per lung in mice that had developed lung metastases. ^△△^*P* < 0.05 vs. injected with scramble siRNA transfected cells plus IL-1β stimulation. **E**: RT-PCR analysis of *p38, MMP2, MMP9* and *c-fos* mRNA expression in the lung metastases of different mice. **F**: IHC analysis of p-p38, MMP2, MMP9 and c-fos protein expression in the lung metastases; IL-1β induced elevated p-p38, MMP2, MMP9 and c-fos protein expression.

At 45 days after injection the cells, all animals (6/6; 100%) in the IL-1β-treated group had developed lung metastases (Figure 7A to D) (group 2; average of metastatic foci in 6 of lung sections was 14 per lung). In contrast, fewer animals (2/6,33.33%) in the control group which were not injected with IL-1β had developed lung metastases (group 1; average of 6 metastases in 6 of lung sections per lung). Whereas, only two animals in the p38 siRNA plus IL-β-treated group developed lung metastases and the number of lung metastases in this group was significantly lower (group 3, average of 4 metastases in 6 of lung sections per lung) and significantly smaller than that of the corresponding group treated with IL-1β (group 2) (Figure 7A to D).

To further confirm whether p38, MMP2 and MMP9 are involved in IL-1β-induced lung metastasis of GA cells, and determine if this process is regulated by AP-1, the mRNA expression levels of *p38, MMP2, MMP9* and *c-fos* (one key component of AP-1) in metastatic lung were quantified by RT-PCR, and p-p38, MMP2, MMP9 and c-fos protein expression in lung sections were examined using IHC. As shown in Figure 7 E and F, the expression levels of p-p38, MMP2, MMP9 and c-fos in the lung metastatic foci were elevated in response to IL-1β. Activation of p38 and the mRNA or protein expression levels of p38, MMP2, MMP9 and c-fos were lower in the metastases formed by the cells transfected with p38 siRNA plus IL-β-treated group (group3) or in the control group (group1) compared to the metastases formed by scramble siRNA plus IL-β-treated group (group 2) (Figure 7E and F). Taken together, the in vivo data further confirms that IL-1β-induced GA cell metastasis is mediated by p38 signaling via AP-1 dependent upregulation of MMP2 and MMP9.

## Discussion

A number of studies have suggested that IL-1β is capable of activating p38 and JNK [11,12], and p38 and JNK play important roles in cancer cell migration and invasion [14,23-26]. Therefore, we hypothesized that IL-1β may contribute to GA cell invasion and metastasis via activating the p38 and JNK pathways. To investigate this possibility, we assessed the ability of IL-1β to activate p38 and JNK, and promote the migration and invasion of GA cells. Our results showed that IL-1β could activate both p38, and JNK, and increase GA cell migration and invasion, and that these effects could be inhibited by p38 siRNA or the p38 inhibitor SB 202190, but not JNK siRNA or JNK inhibitor SP600125. This is the first demonstration that IL-1β can induce GA cell migration and invasion via activation of p38; however, the underlying molecular mechanisms by which IL-β-mediated p38 signaling is regulated during gastric carcinogenesis remain largely unknown.

One potential mechanism by which p38 could increase the invasion and migration of cancer cells is by elevating the levels of MMPs [27]. It is well established that secretion of MMPs with the capacity for extracellular matrix (ECM) degradation is a feature of metastatic cancer cells [28]. MMP2 and MMP9 are two of the most well-characterized MMPs and are closely associated with cancer invasion and metastasis due to their strong proteolytic activity of ECM [29]. We report here also for the first time that the likely molecular mechanism by which IL-1β promotes GA cell migration and invasion may involve the IL-1β/p38/AP-1(c-fos)/MMP2 & MMP9 signaling pathway. We demonstrated that both MMP2 and MMP9 were upregulated in GA cells in response to IL-1β stimulation; these effects were inhibited by siRNAs against *p38, MMP2* or *MMP9*, the p38 inhibitor SB202190, and the MMP2/9 inhibitor BiPs. Furthermore, knockdown of *MMP2* or *MMP9* using siRNAs, or inhibition of MMP2/9 activity using BiPs, significantly decreased IL-1β-induced GA cell migration and invasion. As a serine/threonine protein kinase, p38 is capable of inducing activation of the transcription factor AP-1 [30]. We further found that the IL-1β-induced, p38-mediated upregulation of MMP2 and MMP9 were AP-1-dependent. IL-1β was only able to activate the transcription of *MMP9* promoter regions containing AP-1 sites, and these effects were attenuated by p38 siRNA and the p38 inhibitor SB202190. Additionally, IL-1β-induced activation of AP-1-dependent transcription was inhibited by p38 siRNA.

Phospho-p38 (p-p38), the activated form of p38, could be detected in nearly 50% of the human GA tissue samples tested by IHC assay, and expression of p-p38 was significantly associated with lymph node metastasis, and invasion beyond the serosa in patients with GA. Moreover, the expression of IL-1β positively correlated with the expression of p-p38, MMP2, MMP9 and c-fos in the clinical GA specimens. Furthermore, in vivo data from the metastasis assay demonstrated that the formation of lung metastatic foci by GA cells, and *p38/*p-p38, MMP2, MMP9 and c-fos mRNA and protein expression in the lung metastatic foci were elevated by IL-1β, and reduced by injection of cells transfected with p38 siRNA. Taken together, these data strongly suggest that IL-1β-induced GA cell migration and invasion occur via activation of the p38 signaling pathway which leads to AP-1 activation and upregulation of MMP2 and MMP9. Therefore, p38 plays an essential role in IL-1β-induced metastasis in GA.

JNK is another important MAPK to be well known to play important roles in regulation IL-1β signaling in several different cells [14,31]. However, in this study, JNK was found to be not involved in regulation of IL-1β-induced GA cell migration and invasion. JNK siRNA and JNK inhibitor did not attenuate IL-1β induced GA cell migration and invasion, nor attenuate activation of AP-1 induced by IL-1β. Therefore, IL-1β-promoted GA cell migration and invasion are regulated by p38, but not by JNK.

In summary, we have identified for the first time that IL-1β is functionally involved in the regulation of metastasis in GA via activation of p38. This molecular mechanism involves p38-mediated AP-1-dependent upregulation of both MMP2 and MMP9; and this study strongly suggests that the IL-1β/p38/AP-1(c-fos)/MMP2 & MMP9 pathway may be closely related to metastasis in GA, and therapeutic strategies targeting this pathway may enhance the survival of patients with GA.

## Methods

### Patients and tissue samples

The paraffin embedded blocks from 105 patients with resectable GA who underwent surgery between 2003 and 2005, and pair normal gastric tissues from the same patients were obtained from Fuzhou General Hospital (Fuzhou, Fujian). All of the GA tissue samples chosen in this study were from patients underwent curative gastrectomy with lymph node dissection without surgery related major or serious complications. TNM stages, histological type, and grade of differentiation were identified by several pathologists according to the standards established by NCNN guideline 2011, and no previous benign disease was identified in the samples from patients with metastasis. GA patients were aged 32–84 years old (average 58 years). There were 97 cases with available data of T-stage (92%); T1 (20 cases), T2 (32 cases), T3 (30 cases) and T4 (15 cases). The tissue samples were used with the consent of the patients. This study was approved by the Ethics Committee of Fuzhou General Hospital (Project: 2011-Z032-A).

### Immunohistochemistry for phospho-p38, IL-1β, MMP-2 and 9, and c-fos

To detect the expression of p-p38 in the 105 cases of GA tissues and in nude mice lung metastasic gatric cancer by immunohistochemistry (IHC), we used previously described methods [32-34], with the use of a specific anti-p-p38 antibody (#4631) (1:100 dilution, Cell Signaling Company, Danvers, MA, USA). The assessed standards for staining results were also the same as our previously described for p-Akt2 [33]. Statistical significance was analyzed by the Wilcoxon signed-rank test, Chi-square test, and the Fisher’s exact test. To assess the level of IL-1β, MMP-2 and 9, and c-fos in the tissues mention-above by IHC, we also used the same previous method [32,33]. Anti-MMP-2 (ab110186) and MMP9 (ab38898), and c-fos (ab53036) antibodies used for IHC were 1:250, 1:200 and 1:200 dilution, respectively, and they were from Abcam (Cambridge, MA, USA); Anti-IL-1β antibody was from Santa Cruz (sc-7884) (Santa Cruz, CA, USA) and was diluted 1:100 before use. Spearman’s method was used to analyze the correlation in expression levels of p-p38 with IL-1β, MMP-2 and 9, and c-fos in GA tissue.

### Cell culture and transfection with siRNA

Cell culture and transfection with siRNA were performed in accord with the methods described by us previously [33]. AGS or MKN-45 cells (AGS, American Type Culture Collection, Manassas, VA; MKN-45, Health Service Research Resources Bank, Ibaraki-shi, Osaka, Japan) were grown in F12 or DMEM medium (Invitrogen, Carlsbad, CA, USA) containing 10% fetal bovine serum (FBS) at 37°C in an incubator containing 5% CO_2_. SiRNA against p38 (Cell Signaling), siRNA against JNK (Cell Signaling) or control siRNA (scrambled siRNA) (used as nonsilencing control) (Cell Signaling) and siRNA against MMP-2 or MMP-9 with the targeted position 498 and 2243 of human MMP-2, and targeted position 372 and 1312 of human MMP-9 which were exact the same as the introduction by Luo Y’s [19] (Synthesized in GenePharma Company, Shanghai, China) were transfected into cells, respectively with Lipofectamine 2000 according to the manufacturer’s instructions.

### Western blotting for p38, p-p38, JNK and p-JNK

Western blotting for the expression of p38, p-p38, JNK and p-JNK in AGS or MKN-45 cells was conducted using previously described methods [32,33]. The dilution of primary antibodies used was as followings: rabbit anti-human p38, p-p38, JNK or p-JNK (1:1,000 dilution, Cell Signaling). Anti-β-actin (1:6,000 dilution, Sigma Company) was used as a control for the Western blots.

### Cell migration and invasion assay

For the invasion assay of AGS or MKN-45 cells, we used Sumida T’s and our previous methods [35,36]. Millicell Hanging Cell Invasion Chambers with 8-μm pore filter (Millipore Corporation) were coated with 12 μL of ice-cold Matrigel (Becton Dickinson Labware, Bedford, MA). AGS or MKN-45 cells (5 × 10^4^ per well) were added to the upper chamber of these matrigel chambers in 200 μl serum-free F12 or DMEM medium with or without 20 ng/ml human IL-1β (R & D Systems). Cells were then placed into 24-well plates in F12 or DMEM medium containing 10% FBS. To evaluate the role of the SB202190 or SP600125 or BiPS inhibitor, cells were pre-treated with the reagent for 3 h, and the stimulations were then performed. To evaluate the role of p38 siRNA or JNK siRNA or MMP2 siRNA or MMP9 siRNA or MMP2 siRNA plus MMP9 siRNA in cell migration and invasion, AGS or MKN-45 cells were transfected with scrambled siRNA or p38 siRNA or JNK siRNA or MMP2 siRNA or MMP9 siRNA or MMP2 siRNA plus MMP9 siRNA for 36 h. Following this, the transfected cells were seeded at a density of 5 × 10^4^ per well and then in 200 μl of serum-free medium for the stimulation. When the 20 h incubation was completed, cells were fixed with methanol and stained with Giemsa or crystal violet. Cotton tips were used to remove the cells that remained in the matrigel or attached to the upper side of the filter. Light microscopy was used to count the cells on the lower side of the filter. The assays were performed in duplicate, and the results were then averaged.

The methods used for the migration assay were almost the same as for the invasion assay described above, except no matrigel was used to coat the well and the incubation time was 15 h.

### RT-PCR assay

RT-PCR for amplification of human MMP2, MMP9, c-fos, p38 used the methods described by us previously [36]. Total RNA was extracted from AGS or MKN-45 cells or mouse lung metastatic human gastric cancer cell MKN-45 with the Trizol reagent (Invitrogen). The expression levels of human MMP2, MMP9, c-fos, p38 and GAPDH mRNA were detected by first reverse-transcribing the total RNA, followed by PCR with the following primers: forward: 5′ CCTGATGTCCAGCGAGTG 3′, reverse: 5′ AGCAGCCTAGCCAGTCG 3′ for MMP-2 (295 bp); forward, 5′- CAGTCCACCCTTGTGCTCTTC-3′, reverse, 5′- TGCCACCCGAGTGTAACCAT -3′ for MMP-9 (102 bp); forward: 5′ GTCTCCAGTGCCAACTTCAT 3′, reverse: 5′ CATCTTATTCCTTTCCCTTCG3′ for c-fos (285 bp); forward: 5′ TCCCGTTTGCTGGCTCTT 3′ , reverse: 5′ GGGCACCTCCCAGATTGT 3′ for p38 (442 bp); The expression levels of GAPDH mRNA in each sample were used as controls, and primers used for amplification of GAPDH mRNA were as follows: forward, 5′-GAGTCAACGGATTTGGTCGT-3′, reverse, 5′-TTGATTTTGGAGGGATCTCG-3′ (254 bp).

### MMP-2 and 9 zymography assay

*MMP-2 and 9 zymography assay—*MMP-2 and 9 protease activities in the concentrated supernatant medium of AGS or MKN-45 cells were detected by zymography. Briefly, 8% SDS-PAGE containing gelatin zymogram gels (Applygen Technologies Inc, Beijing, China.) were used to separate the proteins with electrophoresis. Renaturing and developing the gels were performed according to the manufacturer’s instructions, and the gels were then stained with Coomassie blue.

### Immunocytochemical staining and confocal microscopy assay

The relationship between the expression of p-p38, MMP2, and MMP9 in response to IL-1β were detected by immunocytochemical staining and confocal microscopy used the methods described by us [33] instead using anti-p-p38 (Cell signaling), and MMP2 or MMP9 antibody (Abcam).

### AP-1 luciferase reporter gene assay

AP-1 luciferase reporter gene assay were performed. Cells were transfected with AP-1 luc vector (1 μg) or AP-1 plus scramble siRNA or p38 siRNA or JNK siRNA with Lipofectamine2000. B-gal plasmid (containing–galactosidase reporter gene) was co-transfected with AP-1 reporter plasmids to serve as the control for transfection efficiency. Thirty-six hours after transfection, the cells were left untreated or were treated with 20 ng/ml of IL-1β for 12 h. The luciferase assay (for AP-1) and enzyme assay (for B-gal) were then performed according to the instructions of the Promega kit (Madison, WI, USA).

### MMP9-promoter luciferase reporter gene assay

MMP9-promoter luciferase assays were performed as the same methods mentioned-above for AP-1. Cells were transfected by various human MMP9 promoter-luciferase vectors (1 μg) constructed by Genomeditech.com, Shanghai, China, or co-tranfected with scramble siRNA or p38 siRNA with Lipofectamine2000. B-gal plasmid was co-transfected with MMP9 promoter-luciferase plasmids to serve as the control for transfection efficiency. Thirty-six hours after transfection, the cells were left untreated or were treated with 20 ng/ml of IL-1β for 12 h. Luciferase activities were determined using the luciferase assay kit (Promega, Madison, WI) in accordance with the manufacturer’s instruction.

### Invasion assay in nude mice

For the in vivo invasion assay, we followed the protocols described by Yan et al. with minor modifications [37]. Three groups were established; each group contained six mice. Briefly, 2 × 10^6^ MKN-45 cells were injected into the tail vein of 6-week-old male BALB/c nude mice (nu/nu). Group 1 and 2 were injected with MKN-45 cells that had been transfected with a scrambled siRNA; Group 3 was injected with MKN-45 cells that had been transfected with p38 siRNA; group 1 did not receive IL-1β treatment, and group 2 and 3 were treated with IL-1β. The mice were intraperitoneally injected with IL-1β at a concentration of 20 μg/kg/day in 200 μl of PBS for 14 days (one injection every two days), beginning on the day of injection of the MKN-45 cells; the control animals (group 1) were injected with 200 μl of PBS. The mice were euthanized 45 days post-injection of the cells, and the lungs were excised, and subjected to histological analysis under a light microscope after HE staining to determine the extent of metastasis. The total number of metastases per lung was determined by counting the number of metastatic lesions in 6 of lung sections. The methods used for selection of sections and counting the metastases were based on the descriptions by Yan et al [37]. RT-PCR and immunohistochemical analysis of p38 or p-p38, MMP2, MMP9, and c-fos were performed as described above.

### Statistical analysis

Statistical analysis was performed using methods previously described by our laboratory [33,36]. The IHC results were evaluated using the Wilcoxon signed-ranks test, Chi-square test, and the Fisher’s exact test. Spearman’s correlation was used to analyze the relationship between the expression levels of p-p38 and IL-1β, MMP2, MMP9 or c-fos in the GA tissue samples. For the other experiments, all values are expressed as the mean ± SD, and the independent samples *t*-test was performed to determine the significance of the differences between groups. *P*-values < 0.05 were considered statistically significant.

## Competing interests

The authors declare that they have no competing interests.

## Authors’ contributions

QH performed the major experiments, data analysis and wrote the manuscript. FL participated in the design of some of the studies and guided some experiments. XW, JH, YL, YX, XY, HW and LD performed some of the experiments. YY, FX and WL carried out immunohistochemical samples collecting and the results analysis. OX provided clinical data. FZ participated in some experiment design and data analysis. LW and JT contributed to design the study, interpret the data, and funding support. All authors read and approved the final manuscript.
